# A Rare Case of Sudden Death in a Patient with Takotsubo Cardiomyopathy Secondary to Cardiac Rupture

**DOI:** 10.1155/2019/5404365

**Published:** 2019-07-25

**Authors:** Tarun Dalia, Bashar S. Amr, Ankit Agrawal, Archana Gautam, Venkata Rakesh Sethapati, Jared Kvapil

**Affiliations:** ^1^Internal Medicine, University of Kansas Medical Center, Kansas City, KS 66160, USA; ^2^Department of Cardiovascular Medicine, University of Kansas Medical Center, Kansas City, KS 66160, USA; ^3^Internal Medicine Residency Program, Rutgers Robert Wood Johnson Medical School-Saint Peter's University Hospital, New Brunswick, NJ 08901, USA; ^4^Montgomery Internal Medicine Residency Program, University of Alabama, Montgomery, Alabama 36116, USA; ^5^Department of Pathology & Laboratory Medicine, University of Kansas Medical Center, Kansas City, KS 66160, USA

## Abstract

Takotsubo cardiomyopathy (TCM), also known as broken heart syndrome or stress-induced cardiomyopathy, is a rare condition with an estimated incidence of 0.02% of all hospitalizations in United States and 2% of all acute coronary syndrome presentations. TCM predominately presents as a transient wall motion abnormality of the left ventricular apex due to emotional or physical stress. Cardiac rupture in the setting of TCM is an extremely rare phenomenon with limited published case reports. We present a case of a 75-year-old female who had cardiac rupture secondary to TCM and performed a literature review using Ovid MEDLINE for published cases showing this association. After the literature review, we found 20 cases showing this association, which are listed in a tabular fashion.

## 1. Introduction

Takotsubo cardiomyopathy (TCM), also known as stress-induced cardiomyopathy, was first described in Japan in 1990 [1]. It mimics acute coronary syndrome and is characterized by transient systolic and diastolic dysfunction of the left ventricle, wall motion abnormalities, and elevated cardiac biomarkers and is frequently preceded by emotional or physical stress [2]. Ballooning of the left ventricular apex with a finding of patent coronary arteries is typically present on left ventriculography and cardiac catheterization, respectively. It is diagnosed in 0.02% of all nationwide hospitalization and predominantly involves elderly females [3]. The inpatient mortality rate in patients with TCM is 4.5% [3]. There has been a gradual increase in the incidence of TCM with the realization that this entity is not as benign as it was thought to be. As per recent TCM registry, on long-term follow-up, rate of major adverse cardiac and cerebrovascular events was 9.9% per patient-year and death was 5.6% per patient-year [4]. While TCM is usually reversible, it may present with rare complications including systemic embolism, life-threatening ventricular arrhythmias, cardiogenic shock, and cardiac rupture leading to cardiac arrest [5]. We present an exceptional rare case of TCM leading to left ventricular wall rupture. To the best of our knowledge, only 20 cases have been reported so far showing this very rare outcome.

## 2. Case Presentation

**Figure 1:**
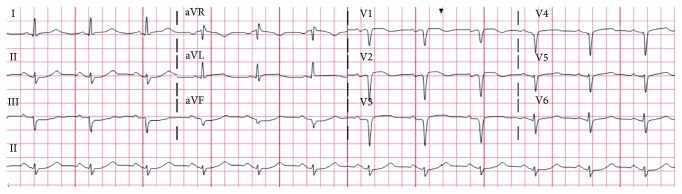
Electrocardiogram showing sinus rhythm at 74 beats/min, left axis deviation, Q waves in V1 to V3, 1 mm ST segment elevation of V2-V3, and poor R wave progression.

**Figure 2:**
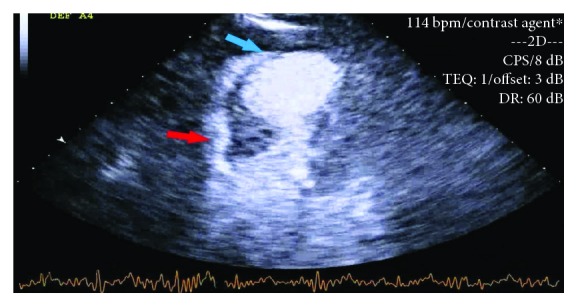
Transthoracic echo showing severely reduced ejection fraction of 30-35%, severe hypokinesis of mid to apical segment with more involvement of the mid anteroseptum, and anterior wall (blue arrow) with basal hyperkinesia and basal asymmetric hypertrophy of the septum (red arrow).

A 75-year-old female with past medical history of coronary artery disease, hypertension, hyperlipidemia, paroxysmal atrial fibrillation, systemic lupus erythematosus, and deep venous thrombosis was transferred from an outside hospital for the management of non-ST segment elevation myocardial infarction (NSTEMI). The patient presented to the local emergency department with substernal chest pain radiating to the left jaw and arm and was found to have an elevated troponin (0.26 ng/ml). She was appropriately given aspirin, heparin, and nitroglycerine and was transferred to our medical centre for further care. On presentation, she was alert and oriented, her blood pressure was 200/100 mmHg, nontachycardic, nontachypneic, and sating at 97-98% on 2 litres per minute of oxygen. Pertinent physical exam findings include bibasilar crackles, no murmurs/rubs, normal heart sounds, and no pedal edema. Pertinent laboratory values include haemoglobin (14.2 gm/dl), platelet count (191 k/*μ*l), white count (14.4 k/*μ*l), INR (1.1), creatinine (0.66 mg/dl), and AST (87 *μ*/l). Electrocardiogram (ECG) on presentation showed sinus rhythm at 74 beats/min, left axis deviation, Q waves in V1 to V3, 1 mm ST segment elevation of V2-V3 (present in prior ECG), and poor R wave progression (Figure 1). A resting 2D Doppler echocardiogram was performed on admission which showed severely reduced ejection fraction of 30-35%, severe hypokinesis of mid to apical segment with more involvement of the mid anteroseptum, and anterior wall with basal hyperkinesia and basal asymmetric hypertrophy of the septum (Figure 2).

The decision was made to undergo urgent cardiac catheterization as her chest pain continued to worsen while on nitroglycerine drip, and the troponin on arrival to our facility was elevated (6.80 ng/ml). While in the elevator, en route to the cardiac catheterization laboratory, the patient became unresponsive. Resuscitation was immediately started, labs at the time of code blue were significant for acute drop in haemoglobin from 14.2 to 6.2, hypokalaemia of 2.5 mmol/l, and arterial blood gas showed metabolic acidosis (pH of 7.27, pCO2 of 34, pO2 of 28, bicarbonate of 15.2 mmol/l). The catheterization team was unable to establish arterial access hence coronary catheterization was not performed. Unfortunately, despite after aggressive resuscitative efforts for 50 minutes, the patient died.

**Figure 3:**
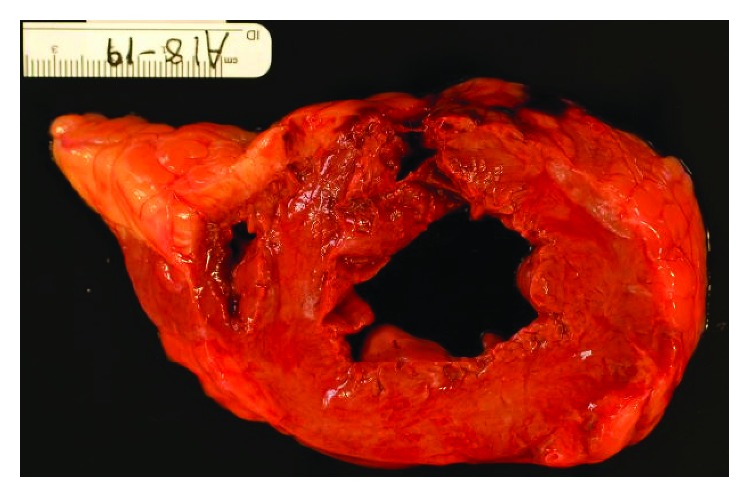
Gross pathology. A cut section of the left ventricle showing an anteriorly located, transmural slit-like rupture (1 cm × 0.8 cm) of the ventricular wall.

**Figure 4:**
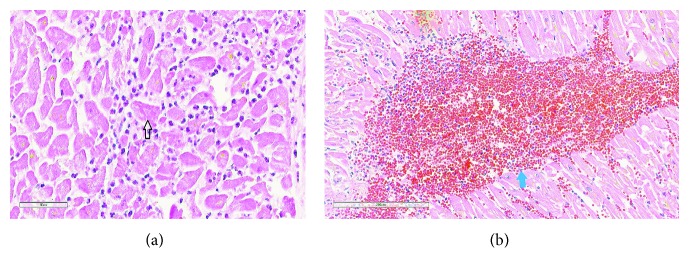
(a) Microscopic pathology. Acute myocardial infarction, H&E, 400x. Note: neutrophilic infiltration of the myocardium, with contraction bands (arrow). (b) Myocardium, H&E, 400x, showing intraventricular haemorrhage (blue arrow).

**Figure 5:**
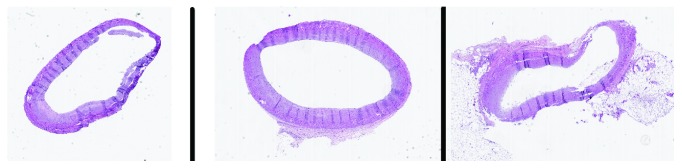
Cut sections of coronary arteries showing patent arteries with minimal atherosclerosis.

Autopsy was performed and revealed 1000 ml of blood in chest wall cavity. Gross pathology revealed a slit-like rupture of 1 cm × 0.8 cm, transmural and located anteriorly 1.5 cm inferior to the base of the heart. The pericardial surface was smooth and tan red in colour (Figure 3). Microscopically, this area had coagulative necrosis, hypereosinophilic appearance of myocytes with abundant ghost cells (cells without nuclei), and cells with pyknotic nuclei. The neutrophilic infiltrate, haemorrhage, and contraction band necrosis can also be visualised (Figure 4). Sacha et al. reported similar rupture site changes indicative of new acute infarct and transmyocardial necrosis leading to rupture in TCM patient [6]. There was epicardial haemorrhage (3.5 cm in length) adjacent to the left anterior descending artery proximal to the site of rupture. Sections in this area revealed area of infarction with early signs of mottling grossly. Interestingly, this area lacked neutrophils suggesting an area of infarction of less than 10 hours. We speculated that this difference in timeline could be due to the discordance of myocardial contraction seen between the apex and base which is often seen in TCM. Most importantly, her major coronary arteries were patent with minimal atherosclerosis and without evidence of organizing thrombus (Figure 5), supporting the histopathological criteria for the diagnosis of TCM.

## 3. Discussion

TCM is generally classified by an acute and profound but reversible left ventricular dysfunction in the absence of significant coronary artery disease. The revised Mayo criteria are most commonly used currently to establish diagnosis of TCM which includes combination of clinical presentation, ECG, transthoracic echo, and angiographic finding [7]. It is often triggered by an acute emotional or physical stress [4]. TCM is associated with a catecholamine surge and adrenoceptor hyperactivity which can increase the cardiotoxicity leading to increased chances of complications. Increased levels of norepinephrine in the state of stress can cause localized ventricular wall motion abnormalities and ST segment changes [8]. In our case, we believe the rupture of the left ventricular wall was multifactorial and a consequence of increased afterload due to elevated blood pressure, probable increased catecholamine surge, and adrenoceptor hyperactivity in the setting of acute stress.

As per Kumar et al., the characteristics of patients with cardiac rupture in TCM when compared to patients with TCM who did not had cardiac rupture include the following: female gender, older age group, higher systolic and diastolic blood pressure, higher frequency of ST elevations in inferior lead, low ejection fraction (EF), and higher left ventricular peak systolic fraction [9]. Our patient portrayed a number of these characteristics including older age, female gender, higher systolic and diastolic pressures, and severely reduced EF.

As histopathological slides from our patient showed no thrombus and minimal atherosclerosis of coronary arteries, our case falls into the newly diagnosed category of MINOCA (myocardial infarction with nonobstructive coronary arteries). TCM is one of the categories of MINOCA; other categories include coronary spasm, coronary dissection, plaque disruption, spontaneous coronary emboli, myocarditis, and coronary microvasculature dysfunction [10]. Our pathological findings were classical for TCM, and apart from the area of rupture, no other area of infarction was seen. Although coronary spasm can also lead to infarction and subsequent death, hence we cannot exclude it definitively, but when our patient's histological slides are taken in consideration with clinical scenario, TCM is the more likely diagnosis.

A thorough literature review of 20 previous cases of TCM complicated with cardiac rupture is listed in Table 1. The characteristics of these patients include 95% females and mean age of 74.9 years. 16 patients presented with chest pain or discomfort (76%); 17 had ST segment elevations (81%); only 9 cases mentioned troponin levels and out of these, 8 showed increased troponin (89%). These findings further support that TCM mimics acute myocardial infarction. Including our case, a total of 17 patients died (81%). The location of rupture was reported in ten cases; six at the apex (60%), two involving the anterior wall (20%), and two involving the posterior wall (20%).

The prognosis of TCM is usually favourable, and left ventricular function improves in the majority of cases with conservative management. Currently, there is no standardised treatment protocol for the patients with TCM [24]. Although beta blockers have been suggested in studies to prevent the progression and recurrence of TCM [16], but recent TCM registry results show no survival benefit with beta blocker [4]. The same registry states that the use of angiotensin receptor blocker or angiotensin-converting enzyme was associated with improved survival [4]. Further studies are needed to delineate the role of beta blockers in TCM. The ST segment elevation in TCM is usually transient and recovers within few days; persistent ST elevation is a warning sign for continued myocardial injury and portend an impending ventricular free wall rupture [24]. The treatment of cardiac rupture in the setting of TCM is surgical repair [11]. It is unclear to date whether anticoagulation plays any role in TCM, but apical thrombus formation had been reported in patients with TCM [8].

## 4. Conclusion

Due to devastating complications of TCM, our case highlights the need for close monitoring of patients with TCM for the first few days. Special consideration should be paid to older female patients as they have higher rates of cardiac rupture.

## Figures and Tables

**Table 1:** Reported cases with cardiac rupture in takotsubo cardiomyopathy patients (Ovid MEDLINE, 2018).

Authors	Age (in years)	Gender	Clinical presentation	EKG finding	Troponin (ng/ml)	ECHO findings	Catheterization findings	Outcomes	Autopsy findings
Kumar et al. [9]	62	Female	Weakness and lightheadedness	ST elevation in I, II, and V5-V6	11.64	EF 30% and severely reduced LV systolic function in mid and distal segments and preserved at basal segments	Nonstentable 50-75% stenosis at mid LAD artery	Death	Slit-like rupture at the mid portion of the posterior ventricular wall
Zalewska-Adamiec et al. [11]	74	Female	Chest pain	Sinus rhythm with QS complex and ST segment elevation in V2-V6	2.041	Contractile disturbances in the apex and hyperkinesis of basal segments with EF 56% and cardiac tamponade	No significant stenosis	Surgical repair and good condition on discharge	Not applicable
Kudaiberdiev et al. [12]	63	Female	Chest pain, lightheadedness, dyspnea	Q waves in III and aVF, T wave inversions in lead II, III, and aVF, and ST-T abnormalities in V5-V6	0.0	LV dilatation EF (35%) moderate MR, hypoakinesia and thinning of LV inferolateral wall with rupture and cross-over blood shunt through two defects into the pericardium	Patent coronary arteries	Surgical repair and good condition on discharge	Not applicable
Sung et al. [13]	73	Female	Chest pain and dyspnea	ST elevation in V2-V5	1.3	Akinesis of mid to apical left ventricle with EF of 58%	Patent coronary arteries	Death	Not performed
Yoshida et al. [5]	78	Female	Chest pain and dyspnea	RBBB and ST elevation in V2-V6 with QS pattern	Not mentioned	Apical kinesis with wall thinning and massive pericardial effusion	Patent coronary arteries	In good condition after discharge	Not applicable
Indorato et al. [1]	70	Female	Chest pain and nausea	Not done	Not done	Not performed	Not performed	Death. Patient died en route to hospital	Hemorrhagic infarction of LV apex. 0.4 cm line of ruptured myocardium from anterior to posterior wall at the apex
Shams [14]	73	Male	Clinical features of pulmonary edema	Sinus tachycardia with Q waves and ST elevation in inferior leads and depression in anterolateral leads	2.840	Left ventriculography: akinesis in the middle and basal-inferior wall and in broad band of mid anterior, mid lateral, and mid septal parts of the left ventricle and hemopericardium. Bedside, limited echo shows cardiac tamponade	Stenosis of all three major arteries. No signs of coronary occlusion	Death	Hemopericardium, perforation of LV free wall at upper posterior part
Kurisu and Inoue [2]	81	Female	Unconsciousness	ST segment elevation in I, II, III, aVF, and V2-V6	Not mentioned	Apical akinesia and basal hyperkinesis	Patent coronary arteries	Death	Not performed
Sacha et al. [6]	81	Female	Chest pain	Diffuse ST elevation in the precordial and limb leads	1.55	Balloon-like LV motion abnormalities with akinesis from mid to apical portions and hyperkinesis of base	No coronary artery disease	Death	Hemopericardium with an LV free wall rupture measuring 10 mm in the apical region and no patent coronary arteries. Inside the heart, there was a mural thrombus in the apical area
Jaguszewski et al. [15]	82	Female	Chest pain	St segment elevation from V1 to V5	14.82	Abnormal LV contraction with apical ballooning pattern with EF of 55%	Patent coronary arteries	Death	Wide penetrating apical rupture as well as 1500 ml of thrombi and liquid blood in the pericardium
Shinozaki et al. [16]	90	Female	Chest pain	ST segment elevation in aVL and V1-V4	Not mentioned	LV apical akinesis and hyperkinesis of base	Intact coronary arteries	Death	Not mentioned
Akashi et al. [8]	70	Female	Chest discomfort	ST elevation in I, II, III, aVL, aVF, and V2-V6	Not mentioned	Apical akinesis and basal hyperkinesis with EF of 51%	Normal coronary arteries	Death	Not performed
Showkathali et al. [17]	86	Female	Chest pain	ST segment elevation in anterolateral and inferior leads	Not mentioned	Shows TCM and no intraventricular gradient	Normal RCA and mild atheromatous LAD artery	Death	Not mentioned
Yamada et al. [18]	71	Female	Shoulder and back pain	St segment elevation in leads V4-V6 and abnormal Q waves in leads V4-V5	Not mentioned	Left ventricular apical wall akinesis. Hyperkinesis in the basal wall with mitral valve systolic anterior wall motion	No coronary artery stenosis	Death	Not performed
Stöllberger et al. [19]	71	Female	Generalized tonic clonic seizure	ST segment elevation in II, II, avF, V5, and V6	Trop-T positive	Left ventricular apical wall, apical septum, and apical posterior wall akinesia and small pericardial effusion	Normal coronary arteries	Death	5 mm left ventricular rupture in the apicoposterior region
Ohara et al. [20]	79	Female	Chest pain	ST segment elevation in 1, aVL, and V1-V5; depression in leads III and avF; and abnormal Q wave in V1-V4	Not mentioned	Akinesis of the left ventricular apical wall	Patent coronary arteries	Death	Rupture in the anterior portion of the left ventricle, patent coronary arteries, and hemopericardium
Mafrici et al. [21]	87	Female	Chest pain and dyspnea	ST segment elevation in inferior leads and V2-V6	Trop-T: 20	Apical dyskinesis with hyperkinesis of left ventricular basal segment	Patent coronary arteries	Death	Not performed
Ishida et al. [22]	67	Female	Chest pain	ST segment elevation in I, avL, and V2-V5	Not mentioned	Apical ballooning, basal hyperkinesis, and left ventricular outflow pressure gradient of 110 mmHg associated with systolic anterior movement of anterior mitral leaflet	Extensive akinesis from the apex to mid portion	Surgical repair to correct the cardiac rupture slit	Not applicable
Leva et al. [23]	65	Female	Chest pain and dyspnea	ST segment elevation in anterior leads	Not mentioned	Akinesis from mid to apical LV and basal hyperkinesis, EF of 30%	No significant stenosis of epicardial coronary arteries	Death	Not mentioned
Iskander et al. [24]	77	Female	Unconsciousness, chest pain, and dyspnea	ST segment. Elevation in leads I, aVL, and V2-V6	Trop-T: 3.60	EF of 25%. Severe dyskinesis of anterolateral wall of LV, no LVOT obstruction	No coronary artery obstruction with slow flow down the LAD	Death	Fresh clot on epicardial surface, slit-like rupture on anteroapical surface of LV
Present case	75	Female	Chest pain and dyspnea	Sinus rhythm, no ST segment elevation, poor R wave progression	6.80	EF of 30-35%, severe hypokinesis of apical LV, and asymmetric hypertrophy of the basal septum	Not performed	Death	Hemopericardium, patent epicardial coronary arteries, slit-like 1 cm × 0.8 cm rupture of the anterior wall of LV

Abbreviations: LV: left ventricle; LAD: left anterior descending artery; RCA: right coronary artery; EF: ejection fraction; TCM, takotsubo cardiomyopathy; LVOT: left ventricular outflow tract obstruction; LAD: left anterior descending artery.
